# Adherence to the Mediterranean Diet and Arterial Blood Pressure in Schoolchildren: The Role of Parental Eating Habits

**DOI:** 10.3390/children12070844

**Published:** 2025-06-26

**Authors:** Alexandra Foscolou, Panos Papandreou, Aikaterini Bikaki, Maria Skouroliakou, Aristea Gioxari

**Affiliations:** 1 Department of Nutritional Science and Dietetics, School of Health Sciences, University of the Peloponnese, Antikalamos, 24100 Kalamata, Greece; alexandra.foscolou@go.uop.gr; 2 Department of Nutrition, IASO Hospital, 37-39 Kifissias Ave., 15123 Athens, Greece; 3 Department of Nutrition & Dietetics, School of Health Sciences and Education, Harokopio University, 70 El. Venizelou Ave., 17676 Athens, Greece; dp4522017@hua.gr (A.B.); mskour@hua.gr (M.S.)

**Keywords:** systolic blood pressure, diastolic blood pressure, Mediterranean Diet, children, parents

## Abstract

**Background/Objectives**: The aim of the present study was to examine the association between the systolic (SBP) as well as diastolic blood pressure (DBP) levels of school-aged children and the adherence of both children and their parents to the Mediterranean Diet. **Methods**: Detailed data on sociodemographic characteristics, eating habits, and anthropometry were collected from each child (N = 102) and their accompanied parent or legal guardian (N = 102). KIDMED (range: −4 to 12) was used to evaluate children’s level of adherence to the Mediterranean Diet and MedDietScore (range: 0–55) was used for parents. Children’s blood pressure (BP) was measured using a validated automated oscillometric device. **Results**: Children with higher adherence to the Mediterranean Diet had significantly lower SBP (*p* < 0.001), DBP (*p* = 0.009), and hip circumference (*p* = 0.01) compared to those with lower adherence. Similarly, children of parents with high adherence to the Mediterranean Diet exhibited lower body weight (*p* = 0.03), waist circumference (*p* = 0.03), SBP (*p* < 0.001), and DBP (*p* = 0.004). In fully adjusted models, for each unit increase in children’s KIDMED scores, a 1.33 unit reduction in SBP (*p* < 0.001), and a 0.77 unit reduction in DBP were found. Similarly, parental MedDietScore was independently associated with significant reductions in children’s SBP (1.01 mmHg) and DBP (0.75 mmHg) (all *p*’s < 0.05). **Conclusions**: Adherence to the Mediterranean dietary pattern by children and parents seems to be associated with lower BP in childhood, supporting the potential value of a family-based approach, particularly involving mothers.

## 1. Introduction

Early childhood provides a critical window of opportunity to establish a foundation for a child’s future and influence the trajectory of their holistic development [1]. Nevertheless, there is evidence that the onset of cardiovascular risk factors, such as increased arterial blood pressure (BP), may occur in early childhood, posing a great public health concern [2,3]. Epidemiological studies reveal that increased BP in school-aged children is prevalent, and many longitudinal studies depict that this phenomenon may lead to hypertension in adulthood along with other complications affecting the heart, blood vessels, and kidneys [4].

From birth onward, exposure to the risk of developing diseases, especially cardiovascular diseases, is high. However, certain modifiable factors, especially those related to daily lifestyle and nutrition, play a key role in shaping long-term health outcomes. Eating habits are established in childhood and are crucial for the primordial and primary prevention of cardiovascular disease risk factors throughout childhood and adolescence into adulthood [5]. Within this context, various dietary patterns have been studied, among which the Mediterranean Diet stands out. The Mediterranean Diet, characterized by an abundance of fruits, vegetables, bread and other grains, potatoes, beans, nuts and seeds, olive oil as a primary fat source, and dairy products, eggs, fish, and poultry in low to moderate amounts [6], has been identified by numerous studies as one of the most effective dietary patterns for the prevention and/or management of cardiovascular diseases [7].

With regard to the adult population, adherence to the Mediterranean Diet mitigates cardiovascular risk factors including anthropometric indices, e.g., body weight, body composition, body circumference, and BP [8]. In fact, many researchers have already associated this dietary pattern with better child cardiometabolic health [9]. However, it must be acknowledged that children’s dietary habits are influenced and directly dependent on their social environment, particularly the home setting [10]. Parents’ dietary habits, the type and quality of food available in the home, and the food preferences as well as level of nutritional literacy of adult family members have a decisive influence on children’s dietary choices [11]. A plethora of studies support the idea that families with healthy eating habits favorably affect their offspring’s nutritional status and overall health, significant similarity being shown between the nutritional patterns of parents and children [12].

Despite this, limited research has directly examined the relationship between parental dietary patterns and health biomarkers in children, such as BP. Even fewer studies simultaneously investigate the combined influence of both children’s and parents’ adherence to the Mediterranean Diet on children’s BP. Thus, this gap in the literature provided the rationale for the current study. The aim of the present study was to examine the association between the systolic (SBP) and diastolic blood pressure (DBP) levels of school-aged children with the adherence of both children and their parents to the Mediterranean Diet.

## 2. Materials and Methods

### 2.1. Study Design and Participants

The current work is a cross-sectional, observational study that enrolled 102 community children and 102 parents from Southern Greece, during the years 2023–2024. Parents or legal guardians were invited to participate in the study through written announcements (posters), electronic invitations, and social media posts by public healthcare centers. A comprehensive information leaflet was distributed to participants who consented to participate, providing the study’s objectives, methods, benefits, and potential risks.

### 2.2. Bioethics

The present study was approved by the School of Health Sciences Research Ethics Committee at the University of Peloponnese (approval no. 249/23-5-2023). The study was carried out following the principles of the Declaration of Helsinki. Participants were informed of the study aims and procedures, and all participants provided written informed consent for study participation prior to enrollment.

### 2.3. Inclusion and Exclusion Criteria

Individuals (children and parents) were eligible for the study if the children were from the community, between 5 and 18 years old, living in Southern Greece, without any chronic, cognitive, or life-threatening diseases, not following special diets or taking nutritional supplements, and not undergoing any medical treatment. Finally, individuals were eligible for the study only if parents or legal guardians consented to both their own and their child’s participation.

### 2.4. Measurements

Detailed data on sociodemographic characteristics, eating habits, and anthropometry were collected from each child and their accompanied parent or legal guardian as described below. The investigators of the study provided practical examples to children and parents/legal guardians when necessary to prevent errors and discrepancies. More specific details are found in the following sections.

#### 2.4.1. Children’s Screening

- Sociodemographic characteristics: Age (years) and gender (female/male).
- Anthropometric indices: A flat scale with light clothing was used to measure body weight, while a calibrated stadiometer with an accuracy of 0.1 cm was used to measure height. The z-scores for BMI-for-age, weight-for-age, and height-for-age were calculated according to WHO growth reference data for boys and girls aged 5 to 19 years old [13]. The International Obesity Task Force (IOTF) established age- and sex-specific body mass index cut-off criteria that were used to evaluate the weight status of the participants [14]. Consequently, they were classified into four categories: underweight, normal weight, overweight, and obese. Waist and hip circumferences were measured with a flexible non-stretch tape without clothes. Waist circumference was measured in the middle, between the lowest rib and the iliac crest, using an inelastic measuring tape to the nearest 0.1 cm, while hip circumference was measured between the greater trochanter and the lower buttock level. To define abdominal adiposity, the waist-to-hip ratio was used.
- Eating habits: The KIDMED score (range from −4 to 12), a quality index intended for children and adolescents, was used to evaluate adherence to the Mediterranean Diet [15]. A score of +1 was assigned to dietary behaviors that show a favorable aspect to this dietary pattern, while a score of −1 was assigned to dietary practices that have unfavorable associations. Lower adherence to the Mediterranean diet was indicated by lower scores, while higher devotion was shown by higher scores.
- Clinical parameters: BP was measured in the right arm by using standard procedures [16]. A validated automated oscillometric device (OMRON M3, HEM-7154-E, Omron Corporation (Kyoto, Japan)) suitable for children was used to measure BP. Using a cuff size chosen based on the child’s mid-upper arm circumference, measurements were taken on the child’s right upper arm. Children were told to sit quietly, with their backs supported, feet flat on the ground, and arms at the level of the heart. Following a minimum of five minutes of rest, two consecutive BP measures were obtained. During the procedure, the children were instructed not to move or speak. The mean value of two BP measurements that were obtained at least two minutes apart was used in the present work [17].

#### 2.4.2. Parental Screening

- Sociodemographic characteristics: The sociodemographic data were age (years), gender (female/male), and level of education (secondary/tertiary/postgraduate education).
- Eating habits: A semi-quantitative, validated, and reproducible food frequency questionnaire was used to evaluate dietary habits [18]. The consumption of several food groups (including red meat and meat products, poultry, fish, milk or other dairy products, fruits, vegetables, legumes, olive oil, and beverages, including alcohol), was assessed. Finally, the level of adherence to the Mediterranean Diet was assessed using the MedDietScore (range: 0–55) [19]. Lower values indicated lower adherence, while higher values indicated greater adherence.

### 2.5. Statistical Analysis

Categorical variables are presented as frequencies (n) and percentages (%), while continuous variables are presented as the mean ± standard deviation (SD) if normally distributed or as the median and interquartile ranges if non-normally distributed. Normality was tested using the Kolmogorov–Smirnov test. Comparisons of continuous variables with level of adherence to the Mediterranean diet (low/medium/high) were performed using analyses of variance (ANOVAs). Associations between categorical variables were tested using the chi-square test. The bivariate Pearson’s rho was used to measure the strength and direction of linear relationships between pairs of continuous variables. Linear regression analyses were used to evaluate the associations between (a) children’s level of adherence to the Mediterranean Diet and children’s SBP or DBP (two different dependent outcomes), and (b) parental level of adherence to the Mediterranean Diet and children’s SBP or DBP. Results from linear regression models are presented as b coefficients and *p*-values. SPSS software (version 29) was used for all calculations (IBM Statistics, Greece).

## 3. Results

**Figure 1:**
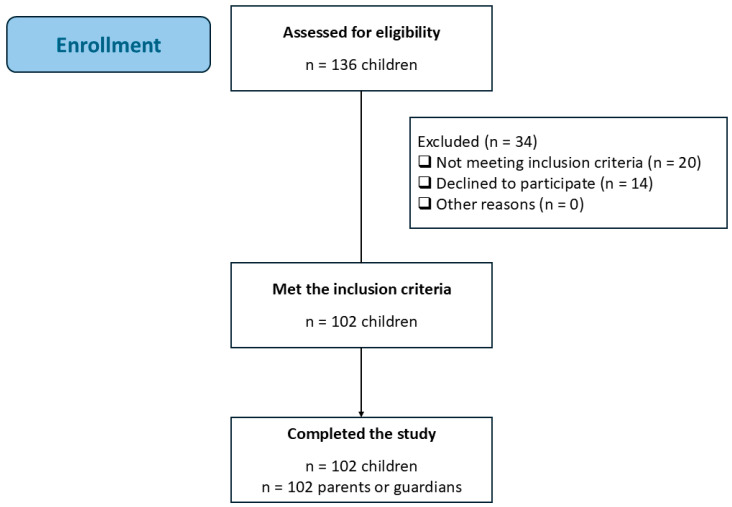
Study flowchart.

As shown in Figure 1, a total of 136 children were assessed to participate in the study.

When study criteria were applied, 20 children were excluded due to seasonal respiratory infections and the presence of chronic conditions (i.e., 2 with diabetes type 1, 3 with growth hormone deficiency). In addition, 14 children dropped out due to lack of communication with their parents or desire to discontinue. Consequently, 102 children and 102 parents (or guardians) completed the study.

Table 1 depicts the participants’ (*N* = 102 children, *N* = 102 parents—one parent for each child) characteristics. The mean age of the children was 12.2 years, with half being of normal weight for their height. They exhibited SBP and DBP levels that were on average within the normal range. As for the parents, apart from 16 individuals, the rest were females. Almost three out of five parents possessed at least tertiary education, exhibited a marginally elevated waist-to-hip ratio, and showed medium adherence to the Mediterranean Diet.

To investigate possible differences in participants’ characteristics depending on the degree of adherence to the Mediterranean Diet, the sample characteristics are presented separately by tertiles of the MedDietScore (Table 2) and the KIDMED score (Table 3).

As shown in Table 2, after children’s stratification regarding their level of adherence to the Mediterranean Diet, it was found that those who adhered the most to the Mediterranean Diet had a relatively smaller hip circumference (*p* = 0.01) and lower SBP (*p* < 0.001) compared to children with a “Low” or “Medium” KIDMED score. Additionally, children with “High” or “Medium” adherence had lower DBP levels than those with “Low” adherence to the Mediterranean Diet (*p* = 0.009). No other statistically significant differences were found in either parents’ or children’s characteristics (all *p*’s > 0.05).

Accordingly, as depicted in Table 3, after stratification regarding the parental level of adherence to the Mediterranean Diet, it was revealed that parents who were characterized as “High” adherents to the Mediterranean Diet had children with a lower weight (*p* = 0.03) and waist circumference (*p* = 0.03), as well as lower SBP (*p* < 0.001) and DBP (*p* = 0.04), compared to children of parents with lower levels of adherence to the Mediterranean Diet.

Since the previous associations between parental or children’s adherence to the Mediterranean Diet and children’s levels of blood pressure may be prone to bias, due to residual confounding, multi-adjusted, nested linear regression models were used to evaluate the research hypotheses (Table 4). Thus, when the association between KIDMED score and SBP or DBP was examined (column A), it was revealed that after adjustments for child’s age, sex, and z-BMI, and parental sex, education level, and MedDietScore (fully adjusted Model 7 for SBP and Model 7 for DBP), for each unit increase in the KIDMED score there was a 1.33 unit reduction in SBP (*p* < 0.001) and a 0.77 unit reduction in DBP (*p* = 0.01). Likewise, in the fully adjusted models (Model 7, column B), for every unit increase in the MedDietScore a 1.01 and 0.75 unit reduction was observed for SBP and DBP, respectively (all *p*’s < 0.001). To further explore this association, additional analyses were performed using the individual food groups of the MedDietScore. It was revealed that parental consumption of wholegrain cereals and lean meat was negatively correlated with children’s SBP (Pearson’s rho coefficient = −0.433, *p* = 0.002 and rho = −0.317, *p* = 0.024, respectively) and DBP (Pearson’s rho coefficient = −0.491, *p* < 0.001 and rho = −0.305, *p* = 0.029, respectively).

## 4. Discussion

This study aimed to investigate the association between child as well as parental Mediterranean Diet adherence level and children’s arterial BP. The findings indicated that higher adherence to the Mediterranean Diet, by both children and parents, was significantly associated with lower SBP and DBP values in children. This association remained significant even after adjustments for children’s age, sex, and z-BMI, and parental sex as well as educational level.

This association is consistent with previous studies showing a protective effect of the Mediterranean Diet on cardiometabolic health in children and adolescents. Indeed, a recent systematic review and meta-analysis of nine randomized controlled trials including 577 children revealed that an increase in Mediterranean Diet adherence was associated with significant reductions in total cholesterol, triglycerides, low-density lipoprotein, and SBP in young individuals [9]. Similarly, results from 197 Spanish adolescents participating in the DADOS longitudinal study also highlighted the importance of Mediterranean Diet promotion to reduce DBP in youth. This association was more pronounced among female children compared to males [20].

As evidenced by the current study, a greater proportion of mothers than fathers participated. It has also been shown in the literature that children of mothers with higher levels of education typically have healthier dietary patterns [21]. Although the present study did not directly assess this association, previous findings, such as those by Bouthoorn et al. [22], who reported significantly lower SBP and DBP values in children of higher-educated mothers, may suggest that maternal education may be an important determinant of a child’s arterial blood pressure. The present study also adds an important new dimension by highlighting parental adherence to the Mediterranean Diet as an independent factor associated with children’s BP. The upbringing and health of children are shaped by the habits and practices of their parents [23], especially mothers [24,25,26].

The negative association between parental adherence to the Mediterranean Diet and BP in children, even after adjusting for factors such as age, gender, BMI, and educational level, suggests the involvement of potential underlying mechanisms. These mechanisms may be both behavioral and environmental. From a behavioral perspective, it should be noted that parents act as role models, influencing children’s food choices, meal frequency and structure, and their general attitudes towards food [27,28]. From an environmental standpoint, parents and legal guardians are those who determine the types of foods that children have access to. Therefore, the nutritional quality of children’s diets is largely dictated by adults [29]. When these food choices align with the Mediterranean Diet pattern, children are more likely to consume nutrient-dense meals, characterized by fewer ultra-processed foods that are often high in sodium and more fiber-rich foods and antioxidants, which have already been linked to decreased BP levels [30].

To the best of our knowledge, this is one of the first studies investigating associations between the eating habits of parents and the clinical outcomes of their children, like BP, in the Greek population. However, the current study has some limitations. Although the level of adherence to the Mediterranean diet was evaluated using validated tools for each group (i.e., KIDMED and MedDietScore), dietary habits, both in parents and children, were assessed based on self-reported food frequency questionnaires, and thus recall bias may exist. Additionally, the relatively small sample size may limit the statistical power to detect significant associations. Furthermore, the study lacked adjustments for potential confounders that could influence blood pressure in children, such as exercise, sleep patterns, sodium intake, or parental history of hypertension. Finally, due to the observational nature of this study, conclusions abouts causes and effects cannot be drawn.

## 5. Conclusions

To conclude, the findings of this cross-sectional study suggest that adherence to the Mediterranean dietary pattern by both children and their parents may be associated with lower BP in childhood. Further prospective studies are required to verify these associations and to gain a more-comprehensive understanding of the underlying mechanisms. Additionally, the examination of this association, along with other cardiometabolic markers, could offer a more comprehensive understanding of the cardiometabolic health of children. Finally, family-based intervention studies may enhance the understanding of how changes in parents’ eating habits and behaviors affect their children. Nonetheless, the present findings underscore the potential importance of adopting a family-centered approach in the early prevention of cardiometabolic risk.

## Figures and Tables

**Table 1:** Sociodemographic, anthropometric, clinical, and dietary characteristics of the participating children (*N* = 102) and parents (*N* = 102).

Children (*N* = 102)	Age (years)	12.2 ± 3.2
	Female, *n* (%)	46 (45)
	Weight (kg)	51.6 ± 19.8
	WAz	0.63 ± 1.2
	Height (cm)	152 ± 19.6
	HAz	0.05 ± 1.3
	Waist circumference (cm)	71.7 ± 11.7
	Hip circumference (cm)	84.6 ± 17.5
	Waist-to-hip ratio	0.89 ± 0.2
	z-BMI	0.62 ± 1.6
	Normal weight, *n* (%)	50 (49)
	KIDMED (−4 to 12)	5.56 ± 2.6
	SBP (mm Hg)	109 ± 12
	DBP (mm Hg)	68 ± 9.1
Parents (*N* = 102)	Female, *n* (%)	86 (84)
	Secondary education, *n* (%)	37 (36.3)
	Tertiary education, *n* (%)	36 (35.3)
	Postgraduate education, *n* (%)	29 (28.4)
	MedDietScore (0–55)	32.0 ± 4.1

Data are presented as the mean ± standard deviation or frequencies (%). WAz: weight-to-age z-score; HAz: height-to-age z-score; BMI: body mass index, SBP: systolic blood pressure; and DBP: diastolic blood pressure.

**Table 2:** Sociodemographic, anthropometric, clinical, and dietary characteristics of the participating children (*N* = 102) and their parents (*N* = 102), based on tertiaries of children’s adherence to the Mediterranean Diet [i.e., KIDMED score (low, medium, or high)].

		First Tertiary Low (n = 19)	Second Tertiary Medium (n = 44)	Third Tertiary High (n = 39)	*p*
Children (*N* = 102)	Age (years)	13.4 ± 3.7	12.4 ± 3.0	11.3 ± 3.3	0.08
	Female, *n* (%)	7 (37)	24 (55)	19 (49)	0.43
	Weight (kg)	59 ± 19	51 ± 18	48 ± 22	0.12
	WAz	0.89 ± 0.9	0.48 ± 1.1	0.67 ± 1.5	0.47
	Height (cm)	159 ± 21	152 ± 18	147 ± 19	0.06
	HAz	0.28 ± 1.3	−0.02 ± 1.1	0.01 ± 1.53	0.70
	Waist circumference (cm)	77 ± 8.4	71 ± 10	70 ± 14	0.10
	Hip circumference (cm)	95 ± 12	84 ± 16	81 ± 19	0.01
	Waist-to-hip ratio	0.83 ± 0.13	0.88 ± 0.21	0.91 ± 0.22	0.39
	z-BMI	0.81 ± 1.3	0.43 ± 1.8	0.75 ± 1.5	0.57
	Normal weight, *n* (%)	8 (42)	25 (57)	17 (44)	0.36
	SBP (mm Hg)	120 ± 11.7	109 ± 8.7	106 ± 13.9	<0.001
	DBP (mm Hg)	73 ± 9.3	66 ± 8.4	67 ± 8.9	0.009
Parents (*N* = 102)	Female, *n* (%)	17 (90)	36 (82)	33 (85)	0.74
	Secondary education, *n* (%)	8 (42)	14 (31)	15 (38)	0.84
	Tertiary education, *n* (%)	7 (37)	17 (39)	12 (31)	
	Postgraduate education, *n* (%)	4 (21)	13 (30)	12 (31)	
	MedDietScore (0–55)	31 ± 4.7	32 ± 4.1	32 ± 3.9	0.49

Data are presented as the mean ± standard deviation or frequencies (%). *p*-values derived from ANOVA for continuous variables or the chi-square test for categorical variables. The level of statistical significance was set at *p* < 0.05. Associations between categorical variables were tested using the chi-square test. WAz: weight-to-age z-score; HAz: height-to-age z-score; BMI: body mass index; SBP: systolic blood pressure; and DBP: diastolic blood pressure.

**Table 3:** Sociodemographic, anthropometric, clinical, and dietary characteristics of the participating children (*N* = 102) and their parents (*N* = 102), based on tertiaries of parental adherence to the Mediterranean Diet [i.e., MedDietScore score (low, medium, or high)].

		First Tertiary Low (n = 31)	Second Tertiary Medium (n = 32)	Third Tertiary High (n = 39)	*p*
Children (*N* = 102)	Age (years)	12.9 ± 3.2	12.4 ± 3.4	11.4 ± 3.3	0.15
	Female, *n* (%)	12 (39)	14 (44)	24 (62)	0.13
	Weight (kg)	58 ± 21	51 ± 20	45 ± 18	0.03
	WAz	0.83 ± 1.4	0.71 ± 1.2	0.41 ± 1.1	0.33
	Height (cm)	154 ± 20	154 ± 18	148 ± 20	0.40
	HAz	−0.16 ± 1.3	0.36 ± 1.37	−0.03 ± 1.2	0.26
	Waist circumference (cm)	78 ± 13	70 ± 13	69 ± 8.3	0.03
	Hip circumference (cm)	91 ± 14	83 ± 22	82 ± 14	0.07
	Waist-to-hip ratio	0.87 ± 0.11	0.91 ± 0.31	0.88 ± 0.15	0.75
	z-BMI	1.1 ± 1.1	0.65 ± 1.2	0.26 ± 2.1	0.12
	Normal weight, *n* (%)	14 (45)	16 (50)	20 (51)	0.81
	SBP (mm Hg)	115 ± 13	109 ± 12	105 ± 10	<0.001
	DBP (mm Hg)	72 ± 8.4	67 ± 8.2	65 ± 9.2	0.004
	KIDMED score (−4 to 12)	5.29 ± 2.6	6.25 ± 2.5	5.21 ± 2.8	0.20
Parents (*N* = 102)	Female, *n* (%)	21 (68)	31 (97)	34 (88)	0.005
	Secondary education, *n* (%)	14 (45)	13 (41)	10 (26)	0.07
	Tertiary education, *n* (%)	11 (35)	10 (31)	15 (38)	
	Postgraduate education, *n* (%)	6 (19)	9 (28)	14 (36)	

Data are presented as the mean ± standard deviation or frequencies (%). *p*-values derived from ANOVA for continuous variables or the chi-square test for categorical variables. WAz: weight-to-age z-score; HAz: height-to-age z-score; BMI: body mass index; SBP: systolic blood pressure; and DBP: diastolic blood pressure.

**Table 4:** Linear regression models evaluating the association of children’s (column A) or parental (column B) level of adherence to the Mediterranean Diet and (a) systolic blood pressure as well as (b) diastolic blood pressure (outcomes) among the 102 children and parents of the study.

		(A) b ± SE, for KIDMED	*p*	(B) b ± SE for MedDietScore	*p*
SBP	Model 1: KIDMED (A) or MedDietScore (B)	−1.70 ± 0.44	<0.001	−1.22 ± 0.28	<0.001
	Model 2: Model 1 + child’s age	−1.22 ± 0.44	0.006	−1.01 ± 0.26	<0.001
	Model 3: Model 2 + child’s sex	−1.23 ± 0.44	0.006	−1.05 ± 0.27	<0.001
	Model 4: Model 3 + child’s z-BMI	−1.35 ± 0.41	0.001	−0.89 ± 0.26	0.001
	Model 5: Model 4 + parents’ sex	−1.32 ± 0.41	0.002	−1.01 ± 0.27	<0.001
	Model 6: Model 5 + parents’ education level	−1.34 ± 0.41	0.002	−1.01 ± 0.27	<0.001
	Model 7: Model 6 + MedDietScore (A) or KIDMED (B)	−1.33 ± 0.39	<0.001	−1.01 ± 0.25	<0.001
DBP	Model 1: KIDMED (A) or MedDietScore (B)	−0.70 ± 0.34	0.04	−0.78 ± 0.21	<0.001
	Model 2: Model 1 + child’s age	−0.65 ± 0.35	0.07	−0.77 ± 0.21	<0.001
	Model 3: Model 2 + child’s sex	−0.67 ± 0.36	0.06	−0.78 ± 0.22	<0.001
	Model 4: Model 3 + child’s z-BMI	−0.71 ± 0.36	0.048	−0.75 ± 0.22	<0.001
	Model 5: Model 4 + parents’ sex	−0.73 ± 0.36	0.044	−0.77 ± 0.23	0.001
	Model 6: Model 5 + parents’ education level	−0.78 ± 0.34	0.024	−0.75 ± 0.21	<0.001
	Model 7: Model 6 + MedDietScore (A) or KIDMED (B)	−0.77 ± 0.32	0.017	−0.75 ± 0.21	<0.001

Data are presented as b-coefficient ± standard error (SE) and *p*-value. SBP: systolic blood pressure; and DBP: diastolic blood pressure.

## Data Availability

Data are unavailable due to privacy or ethical restrictions.

## Abbreviations

The following abbreviations are used in this manuscript:

BMI	Body mass index
DBP	Diastolic blood pressure
SBP	Systolic blood pressure
WAz	Weight-to-age z-score
HAz	Height-to-age z-score
